# Structural Evolution of Water on ZnO(10$\bar{1}$ 0): From Isolated Monomers via Anisotropic H‐Bonded 2D and 3D Structures to Isotropic Multilayers

**DOI:** 10.1002/anie.201910191

**Published:** 2019-10-22

**Authors:** Xiaojuan Yu, Paul Schwarz, Alexei Nefedov, Bernd Meyer, Yuemin Wang, Christof Wöll

**Affiliations:** ^1^ Institute of Functional Interfaces (IFG) Karlsruhe Institute of Technology (KIT) 76344 Eggenstein-Leopoldshafen Germany; ^2^ Interdisciplinary Center for Molecular Materials (ICMM) and Computer-Chemistry-Center (CCC) Friedrich-Alexander University Erlangen-Nürnberg (FAU) 91052 Erlangen Germany

**Keywords:** ab initio molecular dynamics simulations, density functional calculations, hydrogen bonding, IR spectroscopy, water

## Abstract

The surface chemistry of water on zinc oxides is an important topic in catalysis and photocatalysis. Interaction of D_2_O with anisotropic ZnO(10$\bar{1}$ 0) surfaces was studied by IR reflection absorption spectroscopy using s‐ and p‐polarized light incident along different directions. Interpretation of the experimental data is aided using isotopologues and DFT calculations. The presence of numerous species is revealed: intact monomers, a mixed 2D D_2_O/OD adlayer, an anisotropic bilayer, and H‐bonded 3D structures. The isolated water monomers are identified unambiguously at low temperatures. The thermally induced diffusion of water monomers occurs at elevated temperatures, forming dimers that undergo autocatalytic dissociation via proton transfer. Polarization‐ and azimuth‐resolved IR data provide information on the orientation and strength of H‐bonds within the 2D and 3D structures. Ab initio molecular dynamics simulations reveal strong anharmonic couplings within the H‐bond network.

## Graphical abstract

**Hydration of ZnO surfaces**: Comprehensive results from IRRAS and theory lead to a fundamental understanding of the surface chemistry of water on ZnO(10$\bar{1}$ 0). The hydration is initiated by the formation of intact water monomers and proceeds by an autocatalytic dissociation of dimers and 2D adlayers, finally leading to the formation of various H‐bonded 3D structures.

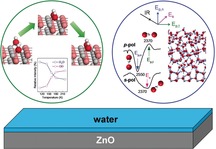

## Introduction

The interaction of water with solid substrates is a topic of pronounced fundamental interest.^1^, ^2^, ^3^ In catalysis, photocatalysis, and corrosion, water is omnipresent, either as a reactant, product, solvent, or contamination.^4^, ^5^, ^6^, ^7^, ^8^, ^9^, ^10^, ^11^, ^12^ In some cases water even takes the role of a catalyst.^13^ The situation is typically very complex, with different types of intermediates being present. Furthermore, the substrate itself may be modified in the process of water adsorption. Achieving an understanding of fundamental processes thus requires combined efforts in a thorough experimental characterization and by theoretical studies. In this context, model systems play a crucial role. Only for precise measurements carried out for well‐defined systems with known structures a meaningful validation of theoretical results can be achieved. A particularly interesting case is zinc oxide (ZnO), which features unique physical and chemical properties.^14^ ZnO can serve either as an active component or as a support material in catalysts and photocatalysts. Hydration processes at ZnO surfaces are of relevance for numerous catalytic reactions, such as methanol production from synthesis gas and the water‐gas shift reaction, producing hydrogen.^15^, ^16^, ^17^, ^18^, ^19^ Previous extensive research efforts combining experiments and density functional theory (DFT) calculations revealed that the properties of water/ZnO systems vary strongly depending on preparation conditions and the surface termination of the substrate.^20^, ^21^, ^22^, ^23^, ^24^, ^25^, ^26^, ^27^, ^28^, ^29^, ^30^, ^31^, ^32^

Despite substantial experimental effort dedicated to water/ZnO interfaces, many important issues are still debated, including the identification of isolated water monomers, the transition from monomer species to a full monolayer, the structural evolution of bi‐ and multilayers, and insight into the importance of the formation of H‐bonded 2D and 3D structures. Progress towards a more detailed understanding of the interaction between water and ZnO is hampered primarily by the lack of reliable reference data recorded for well‐controlled single crystal surfaces using infrared reflection–absorption spectroscopy (IRRAS). Apart from the severe intrinsic experimental difficulties for dielectric substrates,^33^ the extremely weak transition dipole moment of O−H vibrations makes the IR observation of hydroxyl and water species a more challenging task.

Herein, we present a comprehensive atomic‐level picture of the surface chemistry of water on the non‐polar ZnO(10$\bar{1}$ 0) surface derived from experimental data obtained using IRRAS, which are interpreted by state‐of‐the‐art DFT calculations and ab initio molecular dynamics (AIMD) simulations. Our results demonstrate that the interaction of water with ZnO starts with the formation of isolated, intact monomers and then, in the course of increasing coverage, proceeds through a sequence of complex intermediate steps until the multilayer regime is reached. At low coverages, the formation of water dimers is governed by kinetic effects, including thermal diffusion of water monomers and subsequent autocatalytic dissociation. The thorough polarization and azimuth‐ and temperature‐dependent IRRAS data allow for a detailed determination of adsorbate geometry as well as of the orientation and strength of various H‐bonds in 2D and 3D structures.

## Results and Discussion

**Figure 1:**
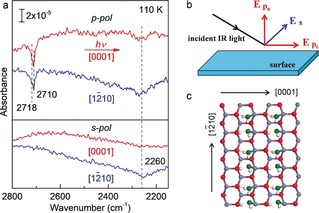
a) Polarization‐ and azimuth‐resolved IRRAS data obtained after saturation adsorption of 1 ML D_2_^16^O on ZnO(10$\bar{1}$ 0) at 110 K. b) Orientation of the *s*‐ and *p*‐polarized components for the incident light. c) DFT‐optimized structure of a water monolayer and the H‐bonds formed on the non‐polar ZnO(10$\bar{1}$ 0) surface. Zn gray, O_s_ red, O_w_ green, H white.

Figure 1 a shows the IRRAS data recorded after adsorption of a saturated D_2_^16^O monolayer (1 ML) on the ZnO(10$\bar{1}$ 0) surface at 110 K by using polarization‐resolved light incident along the two high‐symmetry directions, [0001] and [1$\bar{2}$ 10]. The *p*‐polarized spectra reveal one sharp negative peak at 2710 cm^−1^ with a shoulder at 2718 cm^−1^. Furthermore, a broad negative feature centered at 2260 cm^−1^ is observed. The two high‐frequency vibrations at 2710 and 2718 cm^−1^ are assigned to OD stretching vibrations, while the broad signal centered at 2260 cm^−1^, exhibiting a rather large red‐shift, is characteristic for hydrogen bonded water molecules (ν(O‐D_h_), for a detailed discussion of H‐bond interactions see below). These IR results are in line with DFT calculations that predicted the presence of a stable (2×1) structure consisting of pairs of water molecules, one dissociated and one intact (Figure 1 c).^23^ The (2×1) adlayer was also observed by scanning tunneling microscopy (STM) and He‐atom scattering (HAS).^23^, ^24^

**Figure 2:**
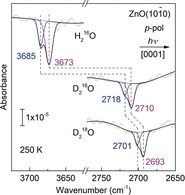
Polarization‐resolved IRRAS spectra recorded after exposing the clean ZnO(10$\bar{1}$ 0) surface to 1ML H_2_^16^O/ D_2_^16^O/ D_2_^18^O at 250 K with *p*‐polarized light incident along the [0001] azimuth. The averaged data were deconvoluted by fitting individual components with Gaussian curves. The blue and magenta lines illustrate H_f_^16^OH (D_f_^16^OD, D_f_^18^OD) and ^16^O_w_H (^16^O_w_D, ^18^O_w_D) species, respectively.

To unambiguously identify the origin of the two hydroxyl vibrational bands, additional isotopic substitution experiments were performed. Figure 2 presents the IRRAS data obtained by exposing the clean ZnO(10$\bar{1}$ 0) surface to H_2_^16^O, D_2_^16^O and D_2_^18^O at 250 K (1 ML), respectively. The IR spectra were recorded using *p*‐polarized light incident along the [0001] azimuth. Again, the spectrum of D_2_^16^O exhibits a major band at 2710 cm^−1^ with a well‐resolved shoulder at 2718 cm^−1^. There is no indication of vibrational signals in the *s*‐polarized spectra (not shown). The IRRAS data for H_2_^16^O shows two IR vibrations at 3673 and 3685 cm^−1^. H‐D isotope shifts of the OH bands with respect to the corresponding OD bands at 2710 and 2718 cm^−1^ provide solid evidence for the assignment of O‐H(D) groups on ZnO(10$\bar{1}$ 0).

The IRRA spectrum recorded for the D_2_^18^O isotopologue allows to gain further insight into the interaction of water with ZnO. As shown in Figure 2, two IR bands are detected at 2701 and 2693 cm^−1^. Both peaks are red‐shifted by 17 cm^−1^ compared to the data for D_2_^16^O. These observations clearly demonstrate that both hydroxyl vibrations are not related to O_s_D groups formed via hydrogen transfer to substrate oxygen ions. Instead, these OD species involve an O atom originating from the water molecule. Accordingly, we assign the predominant band at 2710 cm^−1^ to hydroxyl groups formed via D_2_O dissociation (^16^O_w_D), while the shoulder at 2718 cm^−1^ is ascribed to free, non‐H‐bonded (“dangling”) OD groups of the D_2_O molecules (D_f_^16^OD) pointing away from the surface. The latter must be a minority species at the water cluster boundaries. Furthermore, the absence of sharp IR peaks originating from the O_s_D vibration indicates the formation of H‐bonded O_s_D groups. The interpretation of the IRRAS results is assisted by a thorough theoretical analysis presented below. All experimental and calculated IR bands and their assignments are summarized in Table 1.

**Table 1:** Summary of the experimental and calculated vibrational frequencies for monolayer ^16^OH/^16^OD/^18^OD and H_2_^16^O/D_2_^16^O/D_2_^18^O species adsorbed on the non‐polar mixed‐terminated ZnO(10$\bar{1}$ 0) surface.^[a]^

Adsorbed species	Experimental [cm^−1^]	DFT [cm^−1^]^[b]^
H_f_^16^OH	3685 (+12)	3695 (+7)
D_f_^16^OD	2718 (+8)	2725 (+4)
D_f_^18^OD	2701 (−17)	2708 (−17)
^16^O_w_H	3673	3688
^16^O_w_D	2710	2721
^18^O_w_D	2693 (−17)	2704 (−17)
D_h_^16^OD/^16^O_s_D	2260	2260

[a] The frequency shifts between main peaks and shoulders and the isotope shifts for ^18^O are shown in parentheses. [b] Scaled frequencies (Supporting Information, Table S1).

We have carried out additional experiments using grazing‐emission XPS, which is extremely surface‐sensitive. A quantitative analysis of the O 1s spectra (Supporting Information, Figure S1, and discussion therein) reveals the coexistence of hydroxyl groups (532.0 eV) and intact water (533.0 eV) with a OD/D_2_O content ratio of about 2:1, demonstrating the half‐dissociation of water on ZnO(10$\bar{1}$ 0), in line with the IRRAS results.

The assignment of the vibrational modes is supported by DFT calculations for a series of different structures of water molecules on ZnO(10$\bar{1}$ 0). First, we considered a full water monolayer with the half‐dissociated structure shown in Figure 1 c. The (2×1) surface unit cell contains an O_w_D and an O_s_D group created by the dissociation of a water molecule and an intact D_2_O which forms hydrogen bonds to the O_w_D group and a surface O_s_ atom.^23^, ^24^ For the second and third structures, the water molecules were either both intact or both dissociated within the (2×1) unit cell. According to the DFT results, these two structures are only metastable and slightly higher in energy than the half‐dissociated one.^23^, ^24^ Finally, an isolated water molecule in a large (4×2) unit cell was considered. In this case, only the intact D_2_O molecule is stable. One of the hydrogen atoms of the D_2_O molecule forms a hydrogen bond to a surface O_s_ atom, while the other remains a non‐bonded D_f_ pointing away from the surface.

The calculated OD stretching frequencies for the four structures are listed in Table 1 and the Supporting Information, Table S2. The half‐dissociated structure exhibits only one high‐frequency OD stretching mode for the O_w_D group, which sits almost perpendicular on the surface and does not form a hydrogen bond to an oxygen atom. The calculated frequency of 2721 cm^−1^ is in excellent agreement with the band at 2710 cm^−1^ observed in IRRAS. All other OD stretch vibrations are strongly red‐shifted and are compatible with the experimental broad adsorption band centered at 2260 cm^−1^. For the molecular and the dissociated monolayer, the highest frequency of an OD stretch vibration is located at about 2660 cm^−1^ which cannot account for the experimental high‐frequency bands at 2710 and 2718 cm^−1^. Therefore, these two structures are ruled out.

To capture the impact of the strong anharmonic couplings within the H‐bond network in the monolayer, additional AIMD simulations were performed. The vibrational density of states (VDOS) calculated from the velocity auto‐correlation function for the half‐dissociated water layer are shown in the Supporting Information, Figure S2. Indeed, all OD stretch vibrations of hydrogen atoms involved in hydrogen bonds (D_h_OD and O_s_D) are strongly coupled and can no longer be resolved as individual red‐shifted modes. This finding explains the presence of the experimental broad band centered around 2260 cm^−1^. Only the stretch vibration of the O_w_D group, which sticks out of the water layer, can be seen as individual peak at about 2720 cm^−1^. Overall, the spectrum in the Supporting Information, Figure S2 reproduces all characteristic features of the measured IR spectra.

Finally, for the isolated D_2_O molecule, we find a vibrational mode that is slightly higher in frequency than the O_w_D vibration in the half dissociated monolayer. This confirms the assignment of the experimental shoulder at 2718 cm^−1^ to intact water molecules that do not form a second hydrogen bond to a neighboring water, either because they are sitting isolated on the surface or they are located at the boundary of a water island. The DFT results for water monomers are in excellent agreement with the IRRAS observation shown below.

Hitherto, a direct IR observation of isolated water monomers on metal oxides has not been possible because the IR signals of water at low coverages are very weak. Here, we made an additional effort in instrumentation (for details, see Refs. 33, 34) to obtain such data with extremely high sensitivity and stability and a signal‐to‐noise ratio sufficient to identifying individual peaks reliably. Importantly, the temperature‐resolved IRRAS allows to monitor the thermal diffusion of isolated water molecules, providing detailed insight into the mechanisms of water dissociation on the well‐ordered ZnO(10$\bar{1}$ 0) surface.

**Figure 3:**
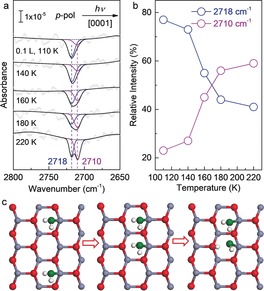
a) Polarization‐resolved IRRA spectra obtained after exposing the clean ZnO(10$\bar{1}$ 0) surface to 0.1 L D_2_^16^O at 110 K and heating gradually to the indicated temperatures. All spectra were measured with *p*‐polarized light incident along the [0001] azimuth at 110 K. The averaged data were deconvoluted by fitting individual components with Gaussian curves. The blue and magenta lines illustrate D_f_^16^OD and ^16^O_w_D species, respectively. b) Relative intensity of OD groups as a function of temperature. c) Ball‐and‐stick model of D_2_O monomer and dimer adsorbed on the non‐polar ZnO(10$\bar{1}$ 0) surface. Zn gray, O_s_ red, O_w_ green, H white.

When the ZnO(10$\bar{1}$ 0) surface was exposed to a small amount of D_2_O (0.1 L) at 110 K, the *p*‐polarized IR spectrum displays one major negative IR band at 2718 cm^−1^ (Figure 3 a). This band is characteristic for intact D_2_^16^O molecules (D_f_^16^OD) and is attributed to the non‐H‐bonded (dangling) O−D vibration excited by the *p*_n,z_‐component of the incident light. This observation reveals the presence of isolated, non‐dissociated water monomers as majority species at 110 K. Upon gently heating the sample, a distinct OD feature at 2710 cm^−1^ grows until it becomes the predominant peak at temperatures higher than 180 K (Figure 3 a). Figure 3 b shows the relative intensity of OD groups as a function of temperature. The 2718 cm^−1^ band decreases from 77 % at 110 K to 44 % at 220 K, while the 2710 cm^−1^ band increases gradually in intensity. As discussed above, the latter band originates from the hydroxyl groups formed via D_2_O dissociation (^16^O_w_D). Again, the coexistence of two distinct OD bands at 2710 and 2718 cm^−1^ points to a partial dissociation of water on ZnO(10$\bar{1}$ 0). Overall, despite the extremely weak signals, the temperature‐resolved IRRAS data confirm the validity of the peak assignment.

These temperature‐induced changes in the IRRAS data reveal that at low temperatures water adsorption first proceeds via the formation of single, intact monomers, in line with the DFT calculations for isolated water species. For temperatures below 110 K the mobility of these species is so low that reactions with adjacent water adsorbates do not occur on the timescale of the experiments. When dimers are formed via thermal diffusion at temperatures above 140 K, the partial dissociation occurs and a proton is transferred to the substrate, yielding two OD species and an adsorbed water molecule [Eq. (1)]:

(1) $$D{}_{2}O+D{}_{2}O+O{}_{s}\to O{}_{w}D+O{}_{s}D+D{}_{2}O$$

In this reaction, the second D_2_O effectively acts as a catalyst. The strong H‐bond interactions between adjacent water adsorbates as well as with substrate O atoms facilitate the hydrogen transfer from water to surface O species (Figure 3 c), in accordance with the theoretical predictions.^35^ This autocatalytic dissociation of water is further supported by the coverage‐dependent IRRAS data at 110 K (Supporting Information, Figure S3), which show an intensity gain of the ^16^O_w_D‐related IR band at 2710 cm^−1^ with increasing the water dose. When a coverage of 1 ML is reached, two bands at 2710 and 2718 cm^−1^ can be resolved, demonstrating the presence of a half dissociated monolayer.

A more thorough characterization of the vibrational bands becomes possible when analyzing the dependence of the IR bands on the polarization and azimuthal direction.^33^, ^36^ Figure 1 a reveals pronounced changes between different IR polarizations as well as for the different high‐symmetry crystallographic directions. For the half‐dissociated water monolayer, the IR spectrum recorded by *p*‐polarized light incident along the [1$\bar{2}$ 10] azimuth (see Figure 1 a) displays an intense negative band at about 2260 cm^−1^, which is attributed to the H‐bonded OD vibration coupling to the *p*_n,z_‐polarized component. For light incident along the [0001] direction, the intensity of this band is much weaker. This could be explained in terms of the offset influence of *p*_n,z_‐polarized and *p*_t,x_‐polarized components (*E*_p,n_ and *E*_p,t_, showing always opposite signs).^37^, ^38^, ^39^, ^40^ The *s*‐polarized light (*E*_s_) is oriented parallel to the surface and perpendicular to the incidence direction. When the incidence plane is aligned along the [1$\bar{2}$ 10] direction, vibrational modes with a transition dipole along the [0001] direction should be excited by *s*‐polarized light. As shown in Figure 1 a, the IR band centered at 2260 cm^−1^ is only detected for *s*‐polarized light incident along the [1$\bar{2}$ 10] azimuth, thus suggesting a strong hydrogen bonding formed along the [0001] direction. Overall, these findings provide direct spectroscopic evidence for a strong H‐bonding between water and substrate O atoms, where the H‐bonds are orientated predominantly along the [0001] azimuth in a tilted configuration (Figure 1 c). Furthermore, the fact that the OD peak at 2710 cm^−1^ (negative sign) was seen only for *p*‐polarized light incident along both the [0001] and [1$\bar{2}$ 10] directions (Figure 1 a) indicates that the hydroxyl species formed via D_2_O dissociation adopt an orientation nearly perpendicular to the substrate surface. These results are fully supported by the DFT calculations.

For the reduced ZnO(10$\bar{1}$ 0) surface, it is known that not O vacancies (F centers) but missing Zn−O dimers are the most characteristic defects,^41^, ^42^ which account for the enhanced reactivity for methanol decomposition, for example.^42^, ^43^ Using the present preparation procedures, we could not identify any defect‐related species in our IRRAS data and, therefore, conclude that the defect density was very low.

After the systematic investigations of coverages in the monolayer regime and below, we now focus on the IRRAS characterization of water bilayer and multilayers, in which the H‐bonding between water molecules also plays a crucial role.

**Figure 4:**
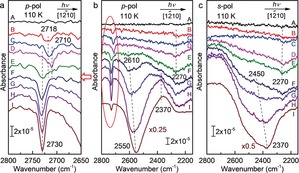
Polarization‐ and azimuth‐resolved IRRA spectra recorded after exposing the clean ZnO(10$\bar{1}$ 0) surface to different doses of D_2_^16^O at 110 K with a),b) *p*‐ and c) *s*‐polarized light incident along the [1$\bar{2}$ 10] azimuth. A) Clean surface and B)–I) exposure to D_2_O: B) 0.1 L, C) 0.2 L, D) 0.5 L, E) 1.0 L, F) 1.5 L, G) 2.0 L, H) 3.0 L, I) 6.0 L.

**Figure 5:**
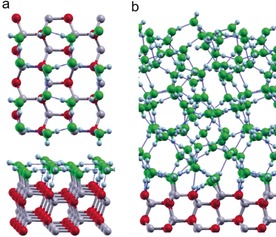
a) DFT‐optimized structure of a water bilayer (top and side views) on the non‐polar, mixed‐terminated ZnO(10$\bar{1}$ 0) surface. b) Snapshot from the AIMD simulation for a water film (side view). Zn  gray, O_s_ red, O_w_ green, H white. H‐bonds are indicated by blue dotted lines.

Figure 4 presents polarization‐dependent IRRAS data obtained after exposing the ZnO(10$\bar{1}$ 0) surface to various amounts of D_2_^16^O at 110 K with *p*‐ and *s*‐polarized light incident along the [1$\bar{2}$ 10] azimuth. Again, isolated water monomers (2718 cm^−1^) are identified as majority species at low coverages (curves B and C), while the 2D half‐dissociated water adlayer (2710 and 2260 cm^−1^) is formed at full monolayer (curve D). New vibrational features appear for coverages beyond a monolayer. After a D_2_O exposure of 1.0 L, the O_w_D‐related band at 2710 cm^−1^ disappears, whereas a weak, red‐shifted signal at 2610 cm^−1^ is detected for *p*‐polarized light. These findings indicate the interconnection with the second layer water molecules via hydrogen bonding along with the bilayer formation. This conclusion is further supported by AIMD simulations. Figure 5 a shows the atomic structure of a water bilayer on the ZnO(10$\bar{1}$ 0) surface. The O_w_D species now form weak H‐bond to the D_2_O in the second layer. The result is a red‐shift of the stretch vibration to about 2600 cm^−1^ in the VDOS (Supporting Information, Figure S5), in excellent agreement with the IR observation.

Apart from the 2610 cm^−1^ band, a high‐frequency OD band appears at 2730 cm^−1^ (Figure 4 a, curve E), which is characteristic for the non‐H‐bonded OD groups of terminal D_2_O molecules. Furthermore, a broad negative feature at 2270 cm^−1^ was observed by both *p*‐ and *s*‐polarized light incident along the [1$\bar{2}$ 10] azimuth (Figure 4). It shifts slightly to higher frequencies and increases in intensity compared to the water monolayer. Importantly, in the corresponding IRRAS data recorded with light incident along the [0001] direction, this band was detected only in the *p*‐polarized light (Supporting Information, Figure S4). These results reveal strong H‐bond interactions between two water layers along the [0001] azimuth, in which the H‐bonds adopt a tilted configuration. Again, there is a good agreement between IR experiments and AIMD simulations (Supporting Information, Figure S5). Based on the calculated bilayer structure (Figure 5 a), every second D_2_O of the second layer forms an H‐bond to a surface O while the second D sticks out of the bilayer into the vacuum. The latter non‐H‐bonded OD vibration gives rise to a new peak in the VDOS slightly below 2700 cm^−1^ (Supporting Information, Figure S5). All other OD vibrations are again strongly coupled via H‐bonding. The center of the band shifts slightly upwards to above 2300 cm^−1^ compared to the water monolayer, in line with the IR results.

Upon exposing to 1.5 L D_2_O at 110 K, water multilayers start to form. Clear evidence for bulk water is the presence of a broad, H‐bond related IR signal centered at about 2600 cm^−1^ coupling with *p*_n,z_‐polarized light (perpendicular to the surface, Figure 4 b) and at about 2450 cm^−1^ coupling with *s*‐polarized light (parallel to the surface along the [0001] direction, Figure 4 c). Compared to the 2260 cm^−1^ band, the large blue‐shift of the thin multilayer IR bands (2600 and 2450 cm^−1^) relative to the monolayers indicates that the intermolecular H‐bond interactions within water multilayers are weaker than those of water with the ZnO(10$\bar{1}$ 0) substrate.

More prolonged exposure to D_2_O (2.0 L to 6.0 L) leads to the formation of thicker D_2_O multilayers. The corresponding polarization‐dependent IRRAS data is shown in Figure 4. The intermolecular H‐bonds parallel to the surface couple with both *p*_t,x_‐polarized light along the [1$\bar{2}$ 10] direction and *s*‐polarized light along the [0001] direction yielding a positive and a negative band centered around 2370 cm^−1^, respectively. These findings reveal the isotropic properties of H‐bonds oriented parallel to the surface within thick water multilayers. The H‐bonds normal to the surface coupling with *p*_n,z_‐polarized light lead to a negative band at 2550 cm^−1^. Compared to the thin D_2_O multilayers where the H‐bond‐related IR bands are centered around 2450 and 2600 cm^−1^, the IRRAS data show clearly a red‐shift to 2370 and 2550 cm^−1^ along with the growth of water multilayers, revealing enhanced intermolecular H‐bond interactions within the 3D water structure. It should be noted that compared to the IR spectra for crystalline ice structures,^44^, ^45^ the broader features observed in Figure 4 are characteristic for the formation of amorphous ice films at low temperatures, in line with the results reported for other water/oxide systems.^46^, ^47^

For water multilayers, there is no substantial difference between spectra recorded with light incident along the [0001] and [1$\bar{2}$ 10] azimuths. As shown in the Supporting Information, Figure S4, different types of H‐bonded OD bands (2600, 2550, 2450, 2370 cm^−1^) were detected by *p*‐ and *s*‐polarized light incident along the [0001] direction, in excellent agreement with the results observed in Figure 4. Again, these results reveal no anisotropic effects for H‐bonds oriented parallel to the surface in thick 3D water structures. This is further confirmed by the structural model of the 3D H‐bond network of water films from the AIMD simulations (Figure 5 b). Furthermore, the calculated VDOS shows strong anharmonic couplings within the H‐bond network, which lead to the formation of a broad band centered slightly above 2400 cm^−1^ (Supporting Information, Figure S6). The unsaturated dangling OD groups at the vacuum interface give rise to a small sharp peak above 2700 cm^−1^. Overall, a good agreement between experiment and theory is obtained.

The temperature‐dependent IRRAS data obtained after multilayer adsorption of D_2_^16^O shows that upon annealing to 150 K the absorption peaks remain unchanged in intensity for both *p*‐ and *s*‐polarized light (Supporting Information, Figure S7). The H‐bonded OD vibrations coupling with *p*_n,z_‐polarized light are red‐shifted from 2550 to 2530 cm^−1^, indicating a slightly enhanced H‐bonding perpendicular to the surface. When heating the sample to 160 K, substantial changes within the spectra are observed. The multilayer D_2_O molecules desorb, resulting in a significant decrease of the intensity as well as in a blue‐shift of the H‐bonded OD vibrations coupling with both *p*‐ and *s*‐polarized light. Increasing the temperature further to 170 K leads to the complete desorption of multilayer water. Finally, upon heating to 180 K, only the half‐dissociated 2D D_2_O adlayer exists on the surface, and two individual OD bands are observed at 2710 and 2718 cm^−1^ (Supporting Information, Figure S7), in full agreement with the results for the water monolayer formed at low temperatures (Figure 4 a).

## Conclusion

In summary, the structural evolution of water on the anisotropic mixed‐terminated ZnO(10$\bar{1}$ 0) surface was investigated over a large range of coverages by polarization‐, azimuth‐, and temperature‐dependent IRRAS in conjunction with DFT calculations and AIMD simulations. The combined results demonstrate that the hydration process is rather complex in nature and is initiated by the formation of intact water monomers. The thermally induced diffusion of isolated water molecules leads to the formation of dimer species in which an autocatalytic dissociation occurs via proton transfer to the substrate. Upon increasing the water coverage, numerous H‐bonded structures were identified, including the well‐ordered 2D OD/D_2_O monolayer, anisotropic water bilayer, and isotropic 3D multilayers. The comprehensive results provide detailed insights into the orientation and strength of H‐bonds within the water 2D and 3D structures.

## Conflict of interest

The authors declare no conflict of interest.

## Supporting information

As a service to our authors and readers, this journal provides supporting information supplied by the authors. Such materials are peer reviewed and may be re‐organized for online delivery, but are not copy‐edited or typeset. Technical support issues arising from supporting information (other than missing files) should be addressed to the authors.

Supplementary
