# Supplementary material for: Structural Evolution of Water on ZnO(101‾ 0): From Isolated Monomers via Anisotropic H‐Bonded 2D and 3D Structures to Isotropic Multilayers
Source: Angew Chem Int Ed Engl. 2019 Oct 22;58(49):17751–7. doi: 10.1002/anie.201910191 (PMC6899783; doi:10.1002/anie.201910191)
Supplement: Supplementary file 1 — Supplementary [file ANIE-58-17751-s001.pdf]

Angewandte Chemie
Eine Zeitschrift der Gesellschaft Deutscher Chemiker

Supporting Information

Structural Evolution of Water on ZnO(10$\bar{1}$0): From Isolated Monomers via Anisotropic H-Bonded 2D and 3D Structures to Isotropic Multilayers

Xiaojuan Yu, Paul Schwarz, Alexei Nefedov, Bernd Meyer, Yuemin Wang, and Christof Wöll**

anie_201910191_sm_miscellaneous_information.pdf

Supporting Information

Structural evolution of water on ZnO($10\bar{1}0$): From isolated monomers via anisotropic H-bonded 2D and 3D structures to isotropic multilayers

Xiaojuan Yu, [a] Paul Schwarz, [b] Alexei Nefedov, [a]
Bernd Meyer, [b] Yuemin Wang,* [a] and Christof Wöll* [a]

[a] *Institute of Functional Interfaces, Karlsruhe Institute of Technology (KIT), 76344 Eggenstein-Leopoldshafen, Germany*

[b] *Interdisciplinary Center for Molecular Materials (ICMM) and Computer-Chemistry-Center (CCC), Friedrich-Alexander University Erlangen-Nürnberg, 91052 Erlangen, Germany*

*Email: yuemin.wang@kit.edu
*Email: christof.woell@kit.edu

1. Methods

IRRAS and XPS experiments. The *in situ* IRRAS experiments were performed in an advanced UHV apparatus, which combines a state-of-the-art FTIR spectrometer (Bruker Vertex 80v) with several other surface-sensitive techniques (XPS and LEED).[1] The innovative design not only allows us to record grazing-incidence IRRAS data for macroscopic oxide single crystal surfaces, but also enables transmission IR experiments on oxide powders supported on an inert metal mesh. This apparatus has been optimized for sensitivity and allows to reliably detect absorbances as low as 1×10^{-5}, a prerequisite for detecting the vibrational signatures of adsorbates on oxide substrates. For a detailed description of the spectrometer see Ref.[1] and Ref.[2].

The ZnO($10\bar{1}0$) surface (10×10 mm^2) was cleaned by a sequence of Ar^+ sputtering (0.5 kV, 3 mA, 1×10^{-6} mbar, 5 min) and annealing (850 K, 5 min) cycles until a high-quality (1×1) LEED pattern was observed. The cleanliness and oxidation states of the sample were monitored by grazing incidence XPS equipped with a VG Scienta R4000 electron energy analyzer. The IRRA spectra were recorded with both *p*- and *s*-polarized light at a fixed grazing incidence angle of 80° along the [0001] and [$1\bar{2}10$] crystallographic directions. Unlike metals, where the so-called surface selection rule states that only vibrational modes with a transition dipole moment (TDM) oriented perpendicular to the surface can be observed,[3] for dielectric substrates also vibrations with a TDM oriented parallel to the substrate can be observed (see Figure S1b). In other words, IR signals can be detected not only for *p*-polarized but also for *s*-polarized light. Note, that both, the *p*- (tangential, $E_{p,t}$) and s-polarized (E_s) components of the incident light, couple to vibrational modes of adsorbed molecules with a TDM component orientated parallel to the surface.[4–7] In addition, in contrast to metals, IRRAS data can show variations when changing the azimuth.

Exposure to H_2O or D_2O was carried out by backfilling the IR chamber through a leak-valve based directional doser connected to a tube (2 mm in diameter) that terminated 3 cm from the sample surface. Less than 1% of other isotopologues were contained in the D_2O. Additional purification of H_2O and D_2O was achieved by repeated cycles of freezing, pumping and thawing. The base pressure during acquisition of IRRAS data was below 1×10^{-10} mbar. Prior to each exposure, a spectrum of the clean ZnO($10\bar{1}0$) surface was recorded as a background reference. All IRRAS data shown here are difference spectra obtained by subtracting the reference. Exposures are given in units of Langmuir (L) (1 L = 1.33×10^{-6} mbar s).

Computational details. The density-functional theory (DFT) calculations for the normal mode vibrational frequencies were carried out with the periodic plane-wave code PWscf of the Quantum Espresso software package,[8] using the Perdew-Burke-Ernzerhof (PBE) exchange-correlation functional,[9] Vanderbilt ultrasoft pseudopotentials,[10] and a plane wave kinetic energy cutoff of 30 Ry. The surface structures were represented by periodically repeated slabs with a thickness of four ZnO double layers and a lateral extension of (2×1) surface unit cells in case of a full water monolayer and bilayer and a (4×2) cell for the single isolated water molecule. The supercells without water thus contained 32 and 128 atoms, respectively. For the k-point sampling a (4,2,1) Monkhorst-Pack mesh was used for the small cell and a (2,1,1) mesh for the large supercell.

All atomic configurations were relaxed with a very tight convergence criterion of 0.001 eV/Å for the residual atomic forces. The atoms in the bottom half of the slab were kept fixed at their bulk positions and only the atoms in the upper half together with the adsorbed water molecules were allowed to move in the geometry optimization. The normal mode vibrational frequencies were calculated in harmonic approximation by a finite difference scheme. The atoms of the adsorbed water molecules and the upper half of the slab were displaced by 0.01 Å in all three Cartesian directions (forward and backward) and the dynamical matrix was determined from the resulting atomic forces, taking the average of the forces from forward and backward displacement. For comparison with experiment, all calculated frequencies were scaled to adjust for the DFT error and to account for deviations due to anharmonic effects. The scaling factors were determined as ratio between the experimental and computed frequencies of the gas phase water molecule, see Table S1.

Table S1. Vibrational frequencies (in cm^{-1}) of the gas phase water molecule. S is the ration between the experimental[11] and the calculated frequencies. The average S_{av} from the symmetric and asymmetric stretch vibration was taken as scaling factor in the subsequent calculations for adsorbed water.

	ν_{bend}(HOH)	ν_s(OH)	ν_{as}(OH)	ν_{bend}(DOD)	ν_s(OD)	ν_{as}(OD)
exp	1595	3657	3756	1178	2671	2788
calc	1574	3732	3847	1152	2690	2819
S	1.0133	0.9799	0.9763	1.0226	0.9929	0.9890
S_{av}		0.9781			0.9910	

The *ab initio* molecular dynamics simulations (AIMD) were carried out with the CPMD software package.[12] The same functional, pseudopotentials and plane-wave kinetic energy cutoff as in the normal mode calculations were used. All simulations were done with the (4×2) supercell. As in the geometry optimizations, the atoms in the bottom half of the slab were kept fixed at their bulk positions. k-point sampling was restricted to the Γ-point.

All structures were equilibrated for 10, 20 and 30 ps using massive Nosé-Hoover thermostats and the Car-Parrinello propagation scheme with a time step of 6 a.u. (about 0.15 fs) and a fictitious electron mass of 600 a.u. For the simulation of the spectra we turned to Born-Oppenheimer MD in the NVE ensemble using a time step of 20 a.u. (about 0.5 fs) and a tight SCF convergence criterion of 10^{-6}. From each of the three equilibration simulations a 100 ps production run was performed. All simulations showed an excellent conservation of the total Hamiltonian energy. The energy drift was always smaller than 6×10^{-8} Ha/ps, with a temperature drift of less than 5 K in the 100 ps runs. The vibrational density of states (VDOS) was calculated from the trajectory using the Fourier transform of the velocity-velocity autocorrelation function. The final spectrum was obtained by averaging the spectra from the three production runs. Simulations were performed for three water coverages: a single monolayer (1 ML), a bilayer (2 ML) and a thick water film consisting of 80 water molecules in the (4×2) supercell. So altogether, we performed about 1 ns of AIMD simulations (three 100 ps trajectories for each of the three structures plus equilibration).

2. Grazing-emission XPS results

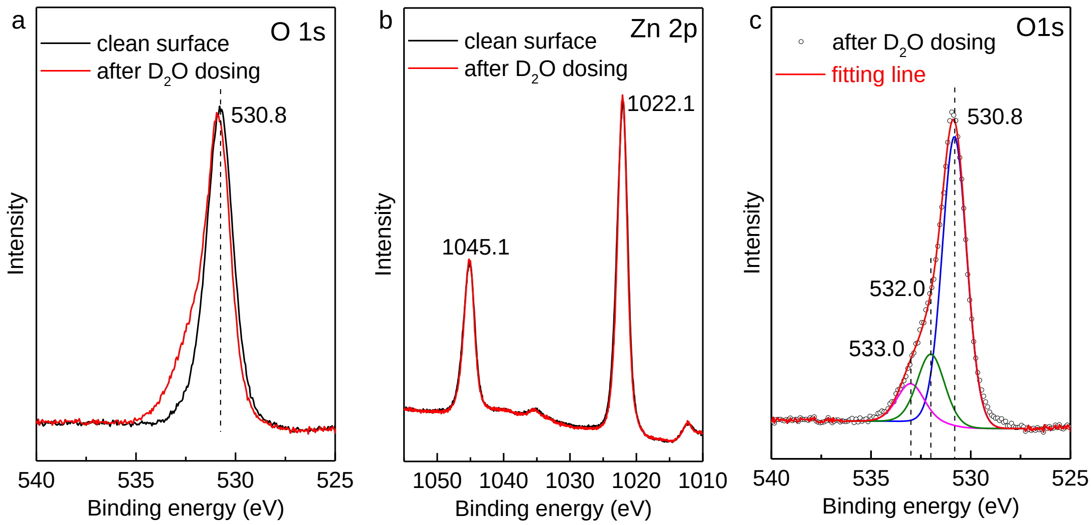

Figure S1. Grazing-emission **a)** O 1s spectra and **b)** Zn 2p spectra recorded before (black curve) and after (red curve) $D_2{}^{16}O$ adsorption on the clean ZnO(10$\bar{1}$0) surface. **c)** corresponding fit of the O 1s spectra after water dosage.

Figure S1 shows the corresponding XPS data of O 1s and Zn 2p spectra for the ZnO(10$\bar{1}$0) surface at 250 K recorded before and after water exposure. For the clean surface, only one O 1s signal at 530.8 eV is observed, which is attributed to lattice O^{2-} ions in the wurtzite structure (Figure S1a).[13] After water adsorption at 250 K, a broad, asymmetric O 1s peak was observed. A deconvolution procedure (fitting of Gaussian after Shirley background subtraction) yielded three components with binding energies of 530.8, 532.0, and 533.0 eV (Figure S1c), which are assigned to substrate O-related, hydroxyl groups of dissociated water molecules on the surface and non-dissociated water molecules, respectively.[14–16] A quantitative analysis reveals that the surface concentration ratio of OD/D_2O amounts to 2:1. Overall, these XPS findings provide additional evidence for the coexistence of hydroxyl and intact waters species, demonstrating the half-dissociation of water on the mixed-terminated ZnO(10$\bar{1}$0) surface, in line with the IRRAS results.

It should be noted that for exposure to water at 250 K the formation of water multilayers can be definitively ruled out since upon water adsorption the total intensity of O 1s as well as Zn 2p shows no substantial changes (Figure S1a and S1b).

3. Results of DFT calculations and MD simulations: isolated monomers and water monolayer

Table S2. Summary of the calculated normal mode vibrational frequencies (in cm^{-1}) for different water monolayer structures and single, isolated water molecules on the nonpolar mixed-terminated ZnO($10\bar{1}0$) surface.

	D_fOD	D_hOD	O_wD	O_sD
1 ML, half-dissociated		2130/2071	2721	2260
1 ML, molecular		2658/2650/1362/1362		
1 ML, dissociated			2662/2654	1494/1429
Single D_2O molecule	2725	1718		

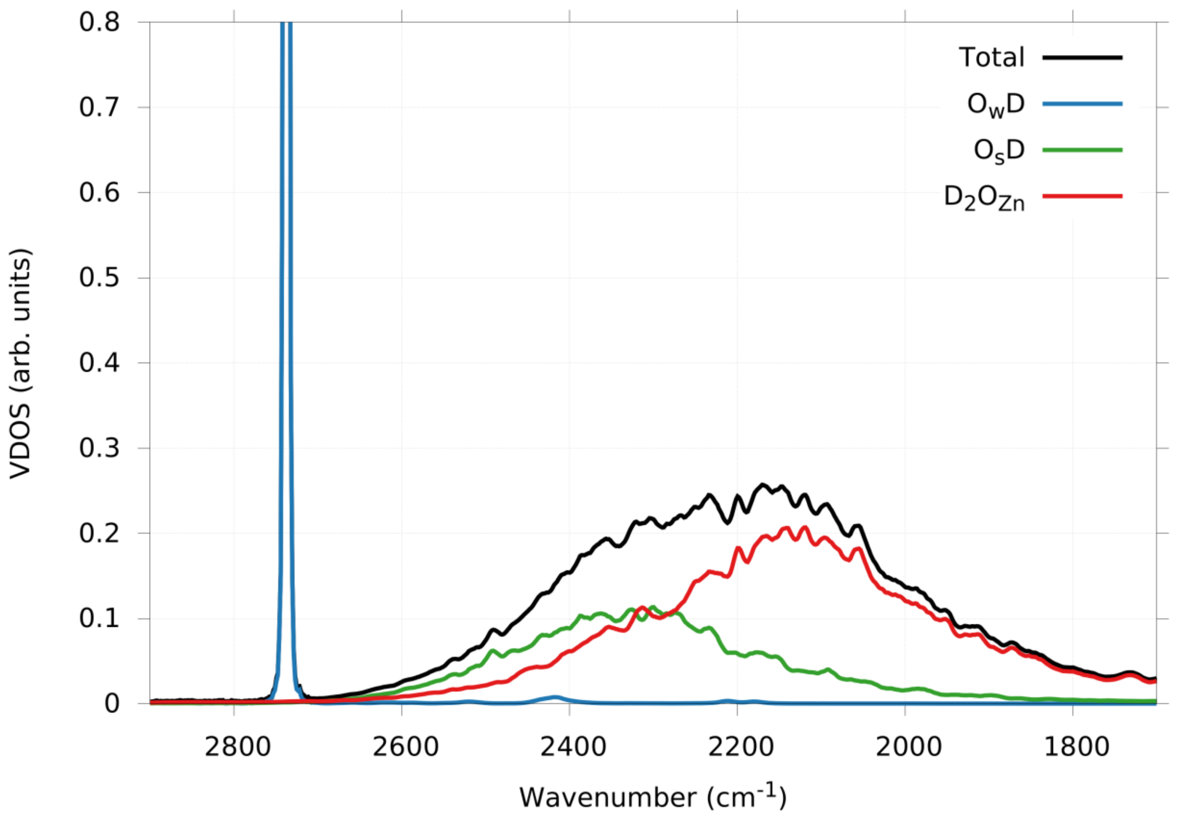

Figure S2. Vibrational density of states (VDOS) from the *ab initio* molecular dynamics simulation for the half-dissociated water monolayer on the nonpolar, mixed-terminated ZnO($10\bar{1}0$) surface. Only the O_wD stretch vibrations give rise to a sharp peak in the VDOS. The stretch vibrations of the O_sD groups and the adsorbed D_2O molecules form a broad band centered around 2200 cm^{-1}.

4. IRRAS results: from water monomer to water multilayers

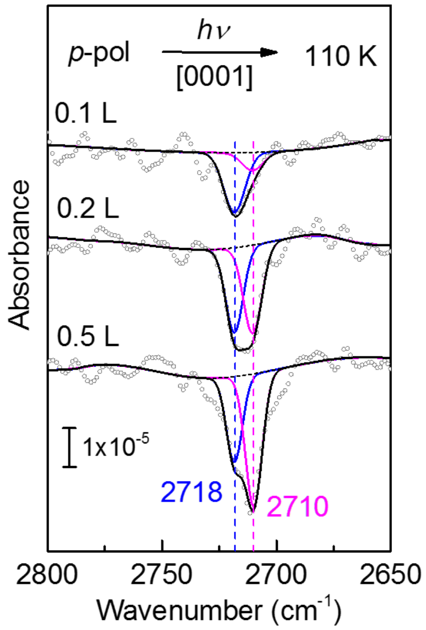

Figure S3. IRRA spectra recorded after exposing the clean ZnO($10\bar{1}0$) surface to different doses of $D_2{}^{16}O$ at 110 K with *p*-polarized light incident along the [0001] azimuth. The averaged data were deconvoluted by fitting individual components with Gaussian curves. The blue and magenta lines illustrate $D_f{}^{16}OD$ and $^{16}O_wD$ species, respectively.

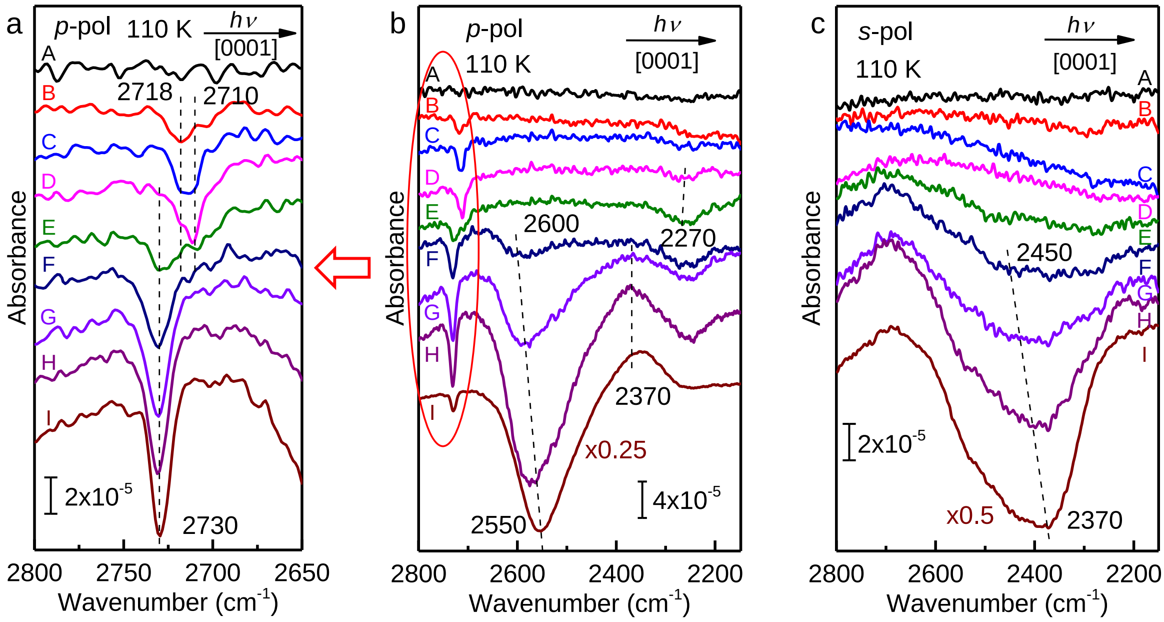

Figure S4. IRRA spectra recorded after exposing the clean ZnO(10$\bar{1}$0) surface to different doses of $D_2{}^{16}O$ at 110 K with **a)** and **b)** *p*- and **c)** *s*-polarized light incident along the [0001] azimuth. (A) Clean surface and (B-I) exposure to D_2O: (B) 0.1 L, (C) 0.2 L, (D) 0.5 L, (E) 1 L, (F) 1.5 L, (G) 2.0 L, (H) 3.0 L, (I) 6.0 L.

5. Results of MD simulations: water bilayer and multilayers

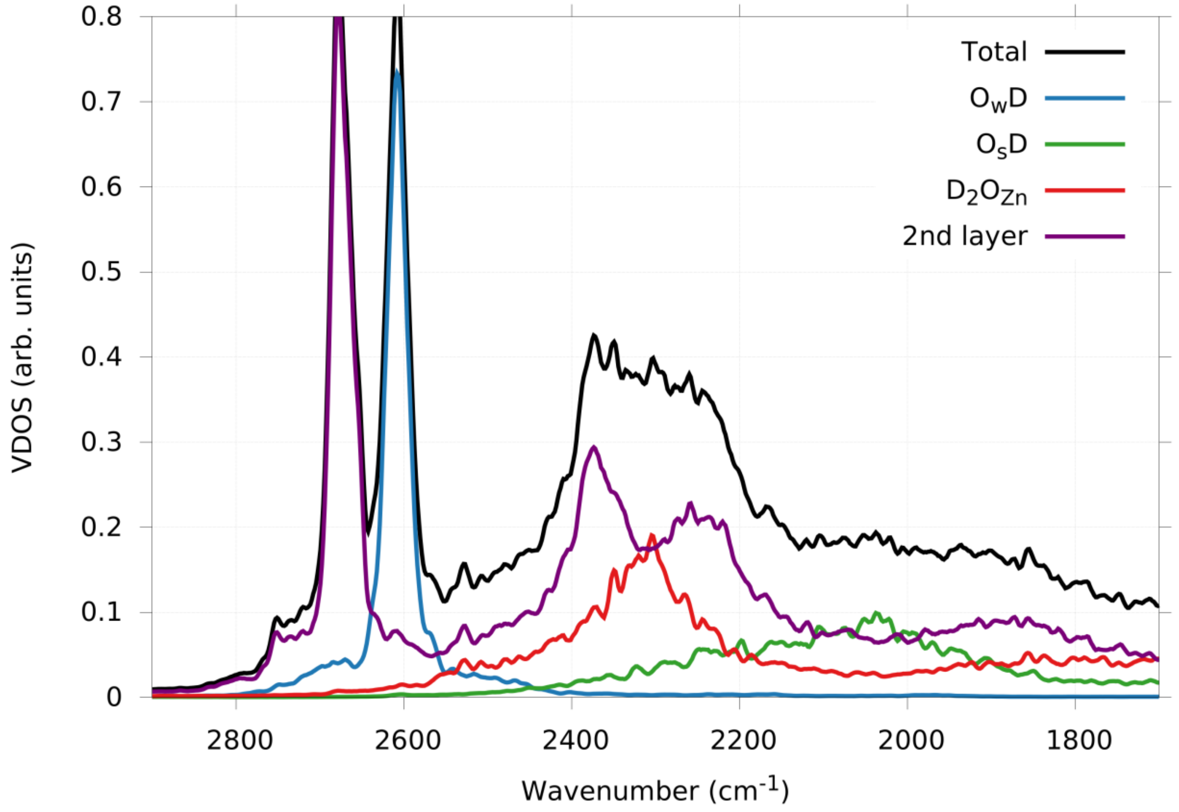

Figure S5. Vibrational density of states (VDOS) from the *ab initio* molecular dynamics simulation for a water bilayer on the nonpolar, mixed-terminated ZnO(10$\bar{1}$0) surface. The O_wD now form a weak H-bond to the D_2O in the second layer. The result is a strong red shift of the stretch vibration to about 2600 cm^{-1}. Every second D_2O of the second layer forms an H-bond to a surface O while the second D sticks out of the bilayer into the vacuum. This second OD vibration gives rise to a new peak in the VDOS slightly below 2700 cm^{-1}. All other OD vibrations are again strongly coupled and form a broad band. The center of the band has shifted upwards to above 2300 cm^{-1}.

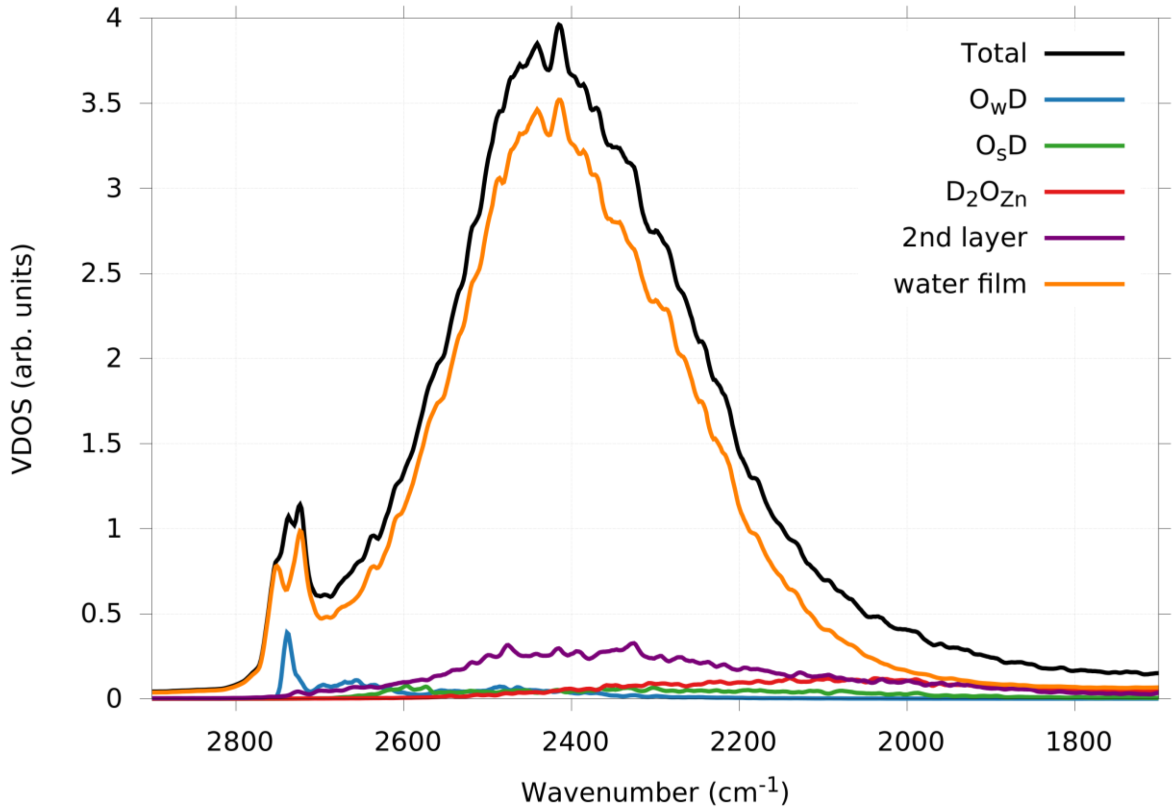

Figure S6. Vibrational density of states from the *ab initio* molecular dynamics simulation for a thick water film on the nonpolar, mixed-terminated ZnO(10$\bar{1}$0) surface. The VDOS is now dominated by the OD stretch vibration of the water molecules in the water film. They form a broad band centered slightly above 2400 cm^{-1}. The unsaturated OD groups (dangling H-bonds) at the vacuum interface give rise to a new small sharp peak above 2700 cm^{-1}.

6. IRRAS results: thermal desorption of water multilayers

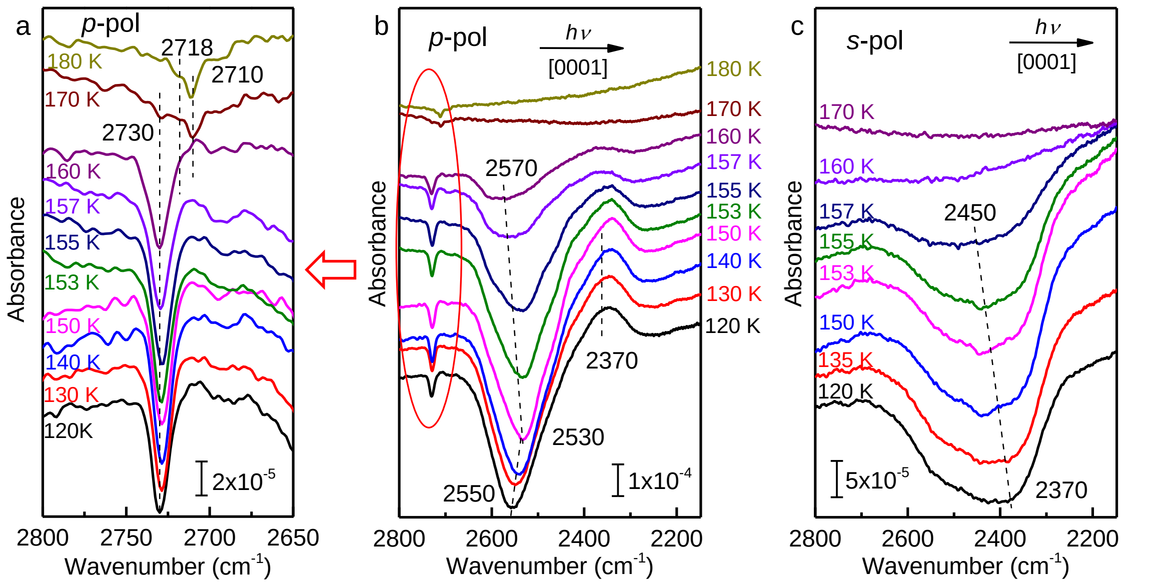

Figure S7. Polarization- and azimuth-resolved IRRA spectra recorded after exposing the clean ZnO($10\bar{1}0$) surface to 5.0 L $D_2{}^{16}O$ at 120 K and heating gradually to the indicated temperatures. All spectra were measured with (a) and (b) *p*- and (c) *s*-polarized light incident along the [0001] azimuth at 120 K.

References

(1) Wang, Y.; Wöll, C. IR Spectroscopic Investigations of Chemical and Photochemical Reactions on Metal Oxides: Bridging the Materials Gap. *Chem. Soc. Rev.* **2017**, *46* (7), 1875–1932.

(2) Yang, C.; Yu, X.; Heißler, S.; Nefedov, A.; Colussi, S.; Llorca, J.; Trovarelli, A.; Wang, Y.; Wöll, C. Surface Faceting and Reconstruction of Ceria Nanoparticles. *Angew. Chemie Int. Ed.* **2017**, *56* (1), 375–379.

(3) Hoffmann, F. Infrared Reflection-Absorption Spectroscopy of Adsorbed Molecules. *Surf. Sci. Rep.* **1983**, *3* (2–3), 107.

(4) Yu, X.; Zhang, Z.; Yang, C.; Bebensee, F.; Heissler, S.; Nefedov, A.; Tang, M.; Ge, Q.; Chen, L.; Kay, B. D., Dohnálek, Z.; Wang, Y.; Wöll, C. Interaction of Formaldehyde with the Rutile TiO_2(110) Surface: A Combined Experimental and Theoretical Study. *J. Phys. Chem. C* **2016**, *120*, 12626–12636.

(5) Buchholz, M.; Weidler, P. G.; Bebensee, F.; Nefedov, A.; Wöll, C. Carbon Dioxide Adsorption on a ZnO($10\bar{1}0$) Substrate Studied by Infrared Reflection Absorption Spectroscopy. *Phys. Chem. Chem. Phys.* **2014**, *16* (4), 1672–1678.

(6) Hansen, W. N. Reflection Spectroscopy of Adsorbed Layers. *Symp. Faraday Soc.* **1970**, *4*, 27.

(7) Mielczarski, J. A.; Yoon, R. H. Fourier Transform Infrared External Reflection Study of Molecular Orientation in Spontaneously Adsorbed Layers on Low-Absorption Substrates. *J. Phys. Chem.* **1989**, *93* (5), 2034–2038.

(8) Giannozzi, P.; Baroni, S.; Bonini, N.; Calandra, M.; Car, R.; Cavazzoni, C.; Ceresoli, D.; Chiarotti, G. L.; Cococcioni, M.; Dabo, I.; Dal Corso, A.; de Gironcoli, S.; Fabris, S.; Fratesi, G.; Gebauer, R.; Gerstmann, U.; Gougoussis, C.; Kokalj, A.; Lazzeri, M.; Martin-Samos, L.; Marzari, N.; Mauri, F.; Mazzarello, R.; Paolini, S.; Pasquarello, A.; Paulatto, L.; Sbraccia, C.; Scandolo, S.; Sclauzero, G.; Seitsonen, A.P.; Smogunov, A.; Umari, P.; Wentzcovitch. R.M. QUANTUM ESPRESSO: A Modular and Open-Source Software Project for Quantum Simulations of Materials. *J. Phys. Condens. Matter* **2009**, *21* (39), 395502.

(9) Perdew, J. P.; Burke, K.; Ernzerhof, M. Generalized Gradient Approximation Made Simple. *Phys. Rev. Lett.* **1996**, *77* (18), 3865–3868.

(10) Vanderbilt, D. Soft Self-Consistent Pseudopotentials in a Generalized Eigenvalue Formalism. *Phys. Rev. B* **1990**, *41* (11), 7892–7895.

(11) *NIST Chemistry Webbook*; Lindstrom, P. J., Mallard, W. G., Eds.; National Institute for Standards and Technology, Gaithersburg, MD, 2001. URL: webbook.nist.gov

(12) Hutter, J. see: http://www.cpmd.org.

(13) Kotsis, K.; Staemmler, V. Ab Initio Calculations of the O1s XPS Spectra of ZnO and Zn Oxo Compounds. *Phys. Chem. Chem. Phys.* **2006**, *8* (13), 1490.

(14) Jeong, Y.; Bae, C.; Kim, D.; Song, K.; Woo, K.; Shin, H.; Cao, G.; Moon, J. Bias-Stress-Stable Solution-Processed Oxide Thin Film Transistors. *ACS Appl. Mater. Interfaces* **2010**, *2* (3), 611–615.

(15) Chen, M.; Wang, X.; Yu, Y. H.; Pei, Z. L.; Bai, X. D.; Sun, C.; Huang, R. F.; Wen, L. S. X-Ray Photoelectron Spectroscopy and Auger Electron Spectroscopy Studies of Al-Doped ZnO Films. *Appl. Surf. Sci.* **2000**, *158* (1–2), 134–140.
(16) Abdel-Wahab, M. S.; Jilani, A.; Yahia, I. S.; Al-Ghamdi, A. A. Enhanced the Photocatalytic Activity of Ni-Doped ZnO Thin Films: Morphological, Optical and XPS Analysis. *Superlattices Microstruct.* **2016**, *94*, 108–118.
